# Spatially Explicit Analyses of Anopheline Mosquitoes Indoor Resting Density: Implications for Malaria Control

**DOI:** 10.1371/journal.pone.0031843

**Published:** 2012-02-14

**Authors:** Colince Kamdem, Caroline Fouet, Joachim Etouna, François-Xavier Etoa, Frédéric Simard, Nora J. Besansky, Carlo Costantini

**Affiliations:** 1 Unité mixte de recherche MIVEGEC (UM1-UM2-CNRS 5290-IRD 224), Institut de Recherche pour le Développement (IRD), Montpellier, France; 2 Laboratoire de Recherche sur le Paludisme, Organisation de Coordination pour la lutte contre les Endémies en Afrique Centrale (OCEAC), Yaoundé, Cameroon; 3 Faculty of Sciences, University of Yaoundé I, Yaoundé, Cameroon; 4 Département de Recherches Géographiques, Institut National de Cartographie (INC), Yaoundé, Cameroon; 5 Eck Institute for Global Health, Department of Biological Sciences, University of Notre Dame, Notre Dame, Indiana, United States of America; Instituto de Higiene e Medicina Tropical, Portugal

## Abstract

**Background:**

The question of sampling and spatial aggregation of malaria vectors is central to vector control efforts and estimates of transmission. Spatial patterns of anopheline populations are complex because mosquitoes' habitats and behaviors are strongly heterogeneous. Analyses of spatially referenced counts provide a powerful approach to delineate complex distribution patterns, and contributions of these methods in the study and control of malaria vectors must be carefully evaluated.

**Methodology/Principal Findings:**

We used correlograms, directional variograms, Local Indicators of Spatial Association (LISA) and the Spatial Analysis by Distance IndicEs (SADIE) to examine spatial patterns of Indoor Resting Densities (IRD) in two dominant malaria vectors sampled with a 5×5 km grid over a 2500 km^2^ area in the forest domain of Cameroon. SADIE analyses revealed that the distribution of *Anopheles gambiae* was different from regular or random, whereas there was no evidence of spatial pattern in *Anopheles funestus* (*Ia* = 1.644, *Pa*<0.05 and *Ia* = 1.464, *Pa*>0.05, respectively). Correlograms and variograms showed significant spatial autocorrelations at small distance lags, and indicated the presence of large clusters of similar values of abundance in *An. gambiae* while *An. funestus* was characterized by smaller clusters. The examination of spatial patterns at a finer spatial scale with SADIE and LISA identified several patches of higher than average IRD (hot spots) and clusters of lower than average IRD (cold spots) for the two species. Significant changes occurred in the overall spatial pattern, spatial trends and clusters when IRDs were aggregated at the house level rather than the locality level. All spatial analyses unveiled scale-dependent patterns that could not be identified by traditional aggregation indices.

**Conclusions/Significance:**

Our study illustrates the importance of spatial analyses in unraveling the complex spatial patterns of malaria vectors, and highlights the potential contributions of these methods in malaria control.

## Introduction

The question of sampling and spatial aggregation of malaria vectors is central to vector control efforts and estimates of transmission [1], [2]. There is significant heterogeneity in the diversity, the abundance and spatial distribution of malaria vectors [3], [4]. Consequently, a thorough knowledge of the spatial patterns of anopheline populations is fundamental for optimal sampling designs and consistent assessments of malaria risk [5], [6], [7], [8]. Moreover, detailed information on the spatial aggregation of malaria vectors has implications for the implementation of cost-effective control strategies at the community level [9], [10], [11], [12].

The analysis of the spatial structure of mosquito populations presents some conceptual and statistical challenges. Numerous methods have been implemented to distinguish among different patterns in the spatial distribution of insects [13], [14]. Traditional methods for count data obtained from a set of locations examine in various ways the relationship between the sample mean and the sample variance [15], [16], [17], [18]. The capacity of these methods to disentangle the spatial patterns is limited because they make no use of information concerning the spatial location of the sample units, and they only infer a degree of non-randomness at an unknown spatial scale [14], [19]. In contrast, new methods were designed in a variety of disciplines to describe and quantify patterns in spatially-referenced count data [20], [21], [22], [23]. Such spatially explicit approaches have attracted growing attention owing to the availability of simple computational tools that can be implemented in Geographic Information Systems (GIS) and in various free software packages [24], [25]. Spatial statistics are commonly used for mapping spatial clusters of diseases, including vector-borne diseases such as malaria, trypanosomes, lymphatic filariasis and arboviral diseases [1]. These methods also have great potential to infer the spatial structure underlying the distribution of a species at a given scale, especially when they are combined with interpretations provided by visualization tools in GIS [22], [26].

However, the implementation of a reliable spatial statistical technique, tailored to suit both fundamental and operational needs, is not always easy. This is due in part to the dearth of data that may provide a framework for taking decisions concerning analytical approaches and interpretations. Several studies have examined distribution patterns in adults of *Culex, Aedes* and *Anopheles* species with one to two spatially explicit tests [27], [28], [29], [30], [31], [32]. Nevertheless, spatial analysis methods and interpretations are more diverse, making the selection complicated. Moreover, each spatial statistic has advantages and limits, and more than one method is usually needed to validate results [33]. More comprehensive explorations of a wider spectrum of spatial analysis tools are therefore necessary to provide cues in the choice of relevant methods for studying patterns of point-referenced counts in mosquitoes across spatial scales.

In this paper, we have used a panel of methods, i.e. correlograms, variograms, Local Indicators of Spatial Association (LISA) and the Spatial Analysis by Distance IndicEs (SADIE), to explore the spatial structure of indoor resting density (hereafter IRD) of two important African malaria vectors mapped across an endemic region of the forest domain of Cameroon. We have compared the distribution patterns provided by spatially-explicit techniques to the dispersion profiles inferred from traditional non-spatial methods. The community-scale entomologic data are usually count data obtained from a number of sampled houses, and aggregated at the locality or village level. This arbitrary assignment of observation units to aggregates may potentially blur some fine-scale structures as spatial analyses are greatly influenced by the scale at which observations are made [34]. In our analyses, we have therefore addressed the effect of scale by comparing dispersion patterns at two aggregation levels: the house or the locality from which mosquito counts were obtained.

## Methods

### Ethics statement

All necessary permits were obtained for the described field studies.

### Study area

**Figure 1:**
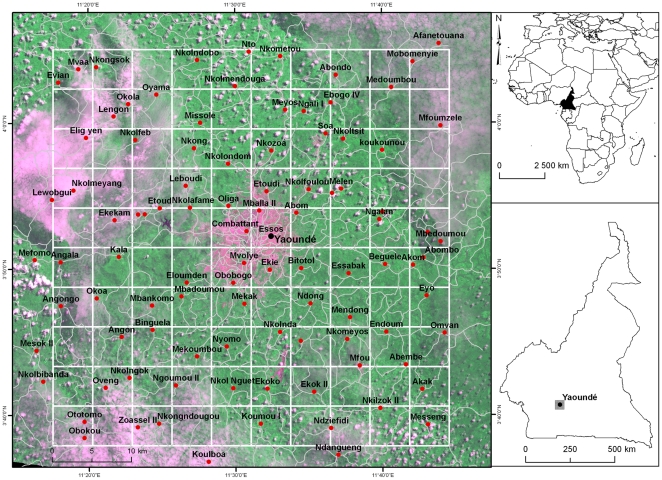
Map showing the study area in Cameroon. The base map is a subset of a Landsat Enhanced Thematic Mapper (ETM+) satellite image with a color composite of red, near-infrared and green bands at 30 m resolution, on which a layer of main roads (in grey) and a 5×5 km grid (in white) are overlaid. In this pseudo-natural image, vegetation appears in shades of green and purple represents deforested areas, bare soils or pixels masked by clouds. The 100 surveyed localities are shown as red circles.

Anopheline mosquito counts came from a 2500 km^2^ area (450–1200 m above sea level), centred on Yaoundé (11°31E, 3°48N), the capital of Cameroon (Figure 1). The region is mainly covered with degraded secondary-growth forest surrounding this major urban centre. The occurrence of many types of anopheline larval habitats explains the presence in the area of several important mosquito vectors maintaining malaria transmission year-round: *An. gambiae sensu stricto*, *An. funestus*, *An. moucheti* and *An. nili* [35], [36], [37].

### Mosquito sampling

The sampling plan was implemented from a map created in ArcGIS 9.2 (ESRI; http://www.esri.com) using a set of geographic data comprising a Landsat Enhanced Thematic Mapper satellite image (1∶60,000) acquired on May 18, 2000; a road map (1∶200,000) and a database of georeferenced human settlements from the National Institute of Cartography of Cameroon (Figure 1). These data layers were chosen to provide regional-scale details of the study area. All the spatial datasets were projected in the UTM 32N zone. We overlaid a 5×5 km grid on the GIS map to divide the study area into 100 isometric cells of 25 km^2^ each. We adopted a spatial resolution of 5 km in light of previous studies on *An. gambiae* dispersal, which estimated a maximum flight distance around 5 km for *An. gambiae* adults [38]. In each cell of the grid, one locality with no less than 100 inhabitants was selected based on accessibility from main roads. Resting mosquitoes were collected in these selected localities between May and July 2007. A few cells of the reference grid were not sampled; a few others presented more than one sampled locality, and six other localities were slightly beyond the borders of the sampling grid (Figure 1). This was due to differences between the initial coordinates provided by the ancillary dataset and the final ground-based geographical coordinates.

As we had limited prior information about mosquito densities in this area [39], we sampled one to six houses per locality with the aim to attain at least 30 individuals per *Anopheles* species in each locality. Female mosquitoes were collected inside human dwellings by spray-sheet catches using aerosol with pyrethrum: a standard procedure for sampling adult *Anopheles* mosquitoes [40]. The geographic coordinates of each sampled house were recorded with a Global Positioning System (GPS) field receiver, and the coordinates of the approximate centroid of the sampled houses were also taken to represent the geographic position of the selected locality. Mosquito specimens were identified following reference morphological identification keys [41], [42]. The geographic and entomologic information were aggregated as point features at two hierarchical levels (house and locality) in an output GIS database using field recorded coordinates. Indoor resting density at the house level represents the number of female mosquitoes of a given species collected in that house divided by the number of sleeping rooms sprayed. At the locality level, indoor resting density of a species is defined as the arithmetic mean of counts per room from all the houses sampled in that locality. To address the effect of the sampling effort (number of houses sampled per locality) on the estimates of IRD, the association between the presence of a species or the number of its specimens captured and the number of rooms sampled was evaluated with correlation tests and logistic regression.

### Non-spatial statistical tests

First, we have examined the patchiness of mosquito counts with two spatially implicit methods (variance-to-mean ratio and Morisita's index of dispersion). Morisita's index is mostly a count-based statistic. Consequently, we have assimilated IRD to counts by rounding the values of densities up to the nearest integer before implementing the two aggregation methods. The ratio (D) between the variance, *s^2^*, and the mean, *m*, of a sampled population provides a simple index for measuring the degree to which individuals are clustered or aggregated within the population [16], [43]. Values of D close to 1 indicate a random dispersion while values of D<1 indicate a uniform dispersion and values of D>1 a clumped dispersion. A one-sided t-test (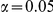), with 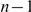 degree of freedom was used to test if D was significantly different from 1 [44].

The Morisita's Index of Dispersion (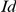) is another statistic commonly used to test if a distribution is random, regular or clumped [15]. Values of 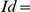
1 indicate a random dispersion while values of 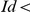
1 indicate a regular dispersion and values of 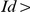
1 a clumped dispersion. We have calculated the Morisita's index on counts, and tested significant deviations from 1 (random) with a chi-square test (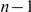 degree of freedom, 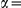
0.05) [44].

### Spatially explicit statistical tests

The choice of spatial statistics to use to infer the spatial patterns with a satisfying degree of reliability in a given dataset is based mainly on the objective of the study, the nature of the data collected and the computational tools available [14], [22], [26]. In exploratory spatial data analysis, statistical tests based on the notion of spatial autocorrelation are the most commonly applied in the examination of spatial patterns of species, but new methods such as SADIE were designed recently in order to circumvent some of the limitations and disadvantages encountered with traditional geostatistical analyses [20]. We have assessed the spatial patterns of IRD at two spatial levels (house and locality) with a set of four spatial analysis tools selected to be representative of the major classes of methods that are widely used to explore the spatial patterns of species [14]. We have used two global methods (correlograms and variograms) and two local methods (LISA and SADIE). Global methods are those that summarize the spatial pattern over the full extent of the study area while local methods are used to detect, to further specify, and to map local patterns and clusters at individual sampling units or at relatively finer spatial scales.

#### Correlograms

The presence of spatial autocorrelation in discrete samples of a continuous variable produces patterns whereby contiguous spatial locations tend to have similar or dissimilar values [13]. This principle has been used to design the Moran's *I* coefficient, an autocorrelation statistic used to study the spatial structure in ecological data [45], [46]:
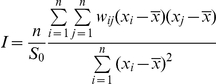
With
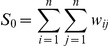
Where *n* indicates the total number of samples; in our case, depending on the geographic level at which the spatial structure was assessed, *n* could be either the number of localities (100) or the total number of houses sampled.

*x_i_* denotes the value of the variable of interest *X* (indoor resting density) at location *I*; *x_j_* represents the value of the same variable at the neighboring location *j* and 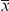 is the sample average of *X*. *w_ij_* is a matrix of spatial weights (connectivity matrix), which defines the degree of spatial interaction across the study region. In general, *w_ij_* = 1 if location *i* and location *j* are neighbors; otherwise, *w_ij_* = 0.

Moran's *I* takes values in the interval [−1, +1]. Positive autocorrelation in the data translates into positive values of *I*; negative autocorrelation produces negative values, and values of *I* close to zero denote absence of autocorrelation [26]. A correlogram is a graph in which autocorrelation values are plotted against the distance lag among sites.

The estimate of spatial autocorrelation can be biased when the data are not normally distributed [22]. Accordingly, indoor resting densities were transformed by a cubic root function to approach a bell shape distribution. The Moran's *I* statistic was calculated with the package *spdep* of the R v.2.9.0 software using the *moran.test* function [47], [48]. Groups of neighboring locations were identified for specified lag distance classes, and the Moran's *I* coefficient calculated for each distance class. Envelopes representing the 95% confidence interval were created around Moran's *I* values with 1000 Monte Carlo randomizations, and statistical significance under the null hypothesis of no spatial autocorrelation was assessed. The smallest lag distance and interclass distance were set at 5 km according to the spatial resolution of the sampling grid we used. We also standardized spatial weights so that all weights summed to unity within a group of neighbors (row standardization). The sum of the weights for a given distance class decreases for large distance classes, and a bias may arise from the fact that only observations at the edge of the sampled population can contribute to the estimates for larger distances. It is therefore customary to limit the description of the spatial structure to half the maximum distance between sampling units [49]: around 35 km in our case.

#### Directional variograms

The directional variogram is another geostatistical tool based on the principle of spatial autocorrelation between sampling units. The variogram or semivariogram is a function relating the variance of a continuous variable to the spatial location of discrete samples [13]:
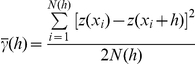
Where 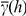 is the estimated variogram value for the distance 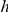, and 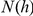 is the number of pairs of points separated by 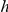. 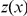 represents the value of the variable at location 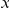 and 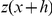 the value of the same variable some 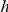 distance away.

Like correlograms, variograms are interpreted graphically, by plotting the estimated variogram 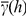 as a function of the distance 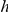. Typically, variogram values are small for low values of 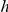, and then increase with increasing distance up to a critical distance where they level off or become constant. Thus, three indices can be used to summarize and interpret the variogram. (1) The *‘sill’* is the value at which the variogram levels off. (2) The *‘range’* represents the distance at which the variogram levels off, defining the average distance below which samples are spatially correlated. (3) The *‘nugget’* is the intercept of the variogram at *h* = 0. Large values of the nugget relative to the sill reveal that most of the spatial structure probably occurs at spatial lags smaller than that of the sample variogram and/or the presence of significant systematic and sampling errors [13], [14]. When the autocorrelation function is the same in all geographic directions considered, the underlying phenomenon is said to be isotropic, whereas its opposite is anisotropy [26]. The effect of anisotropy can be addressed by using directional variograms [22], [26].

Indoor resting densities were transformed with a cubic root function, and directional variograms computed for four spatial directions (0 degree, 45 degrees, 90 degrees and 135 degrees) in the package *geoR* of R, using at least 30 pairs of points for each lag distance class [48], [50]. The maximum lag distance class was limited to half the maximum dimension of the study area (around 35 km). Therefore, variograms were calculated for a total of seven distance classes with an interclass distance of 5 km for each spatial direction. However, at lag 0 (0–5 km), the minimum of 30 pairs of points was not reached for one spatial direction in a few cases. In that circumstance, we did not represent the value of the variogram in that direction at lag 0. Envelopes were created around variograms by taking the maximum and minimum values from 1000 Monte Carlo permutations.

#### Local Indicators of Spatial Association

The Moran's *I* statistic calculated as described above is limited because it measures the spatial clustering only at a global scale, and cannot detect important clusters at local scales or spatial patterns at specific locations. To further evaluate the local clustering, a local version of Moran's *I* can be computed for each spatial unit. The main purpose of this method called “Local Indicators of Spatial Association (LISA)” [23] is to calculate Moran's *I* for each single sampling location, and to generate *p* values in order to assess the statistical significance of these individual indices with a permutation procedure under the null hypothesis of no autocorrelation. To visualize the type and strength of spatial autocorrelation in a data distribution, the local values of Moran's *I* are represented by cluster maps in two ways: a map of *p* values in which the locations of significant spatial clusters are highlighted (*p*<0.01) and a Moran scatter plot. This scatter plot is built from a linear regression between a spatially lagged variable (a variable obtained from the original variable (IRD), by averaging values of observations at neighboring locations of each sampling unit multiplied by their spatial weights) and the original variable. This plot provides indications of the contribution of each sampling unit to the global measure of spatial autocorrelation, and identifies the sampling units that have the greatest influence on the global autocorrelation, based on standard regression diagnostics [23], [51]. The scatter plot also compares values of LISA of individual sampling locations with that of neighboring points, and classifies sampling units by four types of spatial association, each corresponding to one quadrant of the scatter plot. *High/High* are sampling locations with high IRD surrounded by locations that also show high IRD; *High/Low* are locations with high IRD surrounded by locations that have low IRD; *Low/Low* are locations with low IRD surrounded by locations that also have low IRD; *Low/High* are locations with low IRD surrounded by locations that have high IRD.

To create a neighborhood matrix of sampling units, we used the Delaunay triangulation in R. This method is commonly applied to construct neighbors on point features by creating Voronoi triangles [21]. We then calculated the spatial weights for each spatial location, with a row standardization option, in the *spdep* package. Indoor resting densities were transformed by the cubic root function and standardized as suggested in [51]. Local Moran's *I* values were computed on the standardized variable using the *localmoran* function, and a Moran scatter plot was created with the function *moran.plot* in *spdep*. A map displaying spatial locations for which the LISA is significant (*p*<0.01) was generated in ArcGIS.

#### Spatial Analysis by Distance IndicEs (SADIE)

Spatial autocorrelation statistics are not appropriate *sensu stricto* to characterize the spatial patterns of species. The main reason is that, the use of such methods is constrained by strict assumptions of stationarity and normality that are hard to fulfill with species distribution data [20], [22], [26]. These data are, most often, markedly skewed and zero-inflated, and abundance has a non-stationary covariance structure. Moreover, interpretations of Moran's *I* statistics and variograms are jeopardized by the dependence of empirical values on the sampling design [52]. The SADIE technique, by contrast, was designed specifically for clustered ecological count data, and can be fully complementary to traditional geostatistical methods in the exploration of spatial structures of species [20].

This method assimilates the degree of spatial pattern in an observed arrangement of counts to the minimum distance, *D*, that the individuals in the population would need to cover to attain a completely regular arrangement in which abundance is equal in each sample unit [20]. The value of *D* is determined by a transportation algorithm based on the transportation or ‘flow’ of individuals from ‘donor’ sample units with greater than average abundance to ‘receiver’ units with less than average abundance. The value of *D* is calculated for the observed data and for hundreds of simulated datasets generated from randomizations of the observed data. Then, an index of aggregation (*I*a) is computed by dividing the distance to regularity (*D*) from the observed data to the mean *D* over the randomizations. SADIE analyses derive this aggregation index together with a probability *P*a for formal statistical tests of randomness under the null hypothesis of spatial randomness. *P*a denotes the proportion of permutations with distance to regularity less than or equal to the observed value. For a given dataset, values of *Ia*>1 usually indicate an aggregated sample; *Ia* = 1 is expected for spatially random data, and *Ia*<1 denotes a regularly dispersed sample.

The SADIE method also describes the degree of clustering in count data. The term “cluster” is used to mean a region of either relatively large counts close to one another (i.e. a patch) or of relatively small counts (i.e. a gap) in two-dimensional space [20], [53]. For each sampling unit, a clustering index is calculated, measuring the degree to which the unit contributes to clustering as a member of a group of donor units that constitute to a patch (v_i_, positive values), or as a member of a group of receiver units that contribute to a gap (v_j_, negative values). The mean of these clustering indices (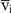,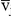) are computed from randomizations together with associated probabilities (*P_i_* and *P_j_*) to test the statistical significance of these clustering indices under the null hypothesis of a random distribution. Values of clustering indices around unity indicate that the data conform to the null hypothesis of spatial randomness. A value of at least one index well above 1 indicates some form of spatial non-randomness. When plotted on a map of the sampling units, the values of the indices, v_i_ and v_j_, indicate the location and extent of clusters in the data. For a particular dataset, a patch cluster or a gap cluster is defined as a set of neighboring units for which the value of the unit clustering index is greater than an arbitrary threshold (generally +1.5 for v_i_ and −1.5 for v_j_). The values of the indices may also be displayed on a map of sample units by a contour plot showing the exact dimension and area covered by patch and gap clusters. SADIE is designed specifically for count data. Accordingly, values of indoor resting density were rounded up to the nearest integer before SADIE analyses that were carried out in SADIEShell 1.22 (http://www.rothamsted.ac.uk/pie/sadie/SADIE_downloads_software_page_5_2.htm). Contour plots were generated in the package SURFER 9.1.352 by spatial interpolation between sample units with the kriging method. We used the highest number of randomization (5957) and the non parametric approach to account for the skewness in the species distribution data.

## Results

### Distribution and abundance of malaria vector species

**Figure 2:**
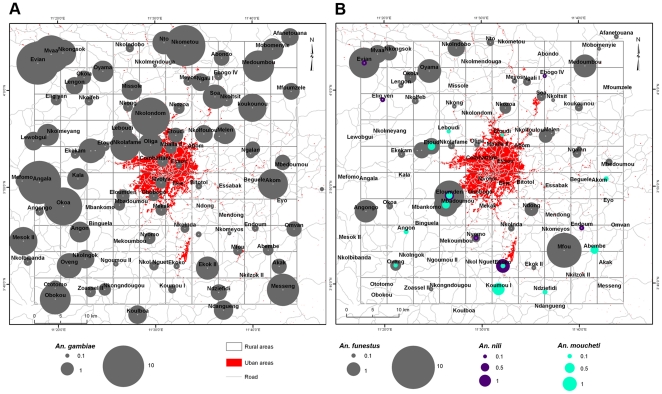
Distribution of indoor resting densities of *An. gambiae* (A) and other malaria vectors (B).

A total of 310 houses and 780 sleeping rooms were sampled during the study period. The mean ± standard error was 3.1±0.90 for the number of houses and 7.8±2.99 for the number of rooms sampled per locality. The total number of houses visited per locality ranged from 1 to 5 while the total number of rooms varied between 2 and 18. In three of the 100 sampled localities, mosquito collections took place in only one house: Abom (2 rooms), Evian (5 rooms) and Mfou (4 rooms). Five malaria vectors species (*An. gambiae sensu lato*, *An. funestus*, *An. moucheti*, *An. nili* and *An. hancocki*) were found in human dwellings in this area. *An. gambiae* was the most widespread species, present in 88 cells of the sampling grid, followed by *An. funestus* which was found in 47 localities (Table 1 and Figure 2). The three other vectors (*An. moucheti*, *An. nili* and *An. hancocki*) were distributed locally, and their presence was observed respectively in 12, 8 and 4 localities of the sampling grid (Figure 2). *An. gambiae* was also the most abundant species with a total of 1313 specimens captured, accounting for 78% of *Anopheles* collected. The second most abundant species was *An. funestus* with a total of 327 individuals captured, representing 19% of all *Anopheles* specimens sampled. Only 4 individuals of *An. hancocki* were obtained in four different cells. The average number of mosquitoes, resting in one room, varied between species as well: the 10^th^ and 90^th^ percentiles of the average indoor resting densities ranged from [0,0] for *An. moucheti* and *An. nili* to [0,5] for *An. gambiae* (Table 1).

**Table 1:** Distribution and abundance of malaria vectors in the study area.

Species	Total	Cells occupied	Average IRD	95% CI IRD	10th centile IRD	90th centile IRD
***An. gambiae***	1313	88	1.94	[1.45–2.43]	0	5.00
***An. funestus***	327	47	0.41	[0.18–0.64]	0	1.00
***An. moucheti***	22	12	0.01	[−0.01–0.03]	0	0.00
***An. nili***	23	8	0.02	[−0.01–0.05]	0	0.00

### Non-spatial tests of spatial aggregation

We examined the spatial structure only in *Anopheles* species present in more than 20% of the cells of the sampling grid (*An. gambiae* and *An. funestus*). First, we have tested how far the uneven sampling we have performed was confounding the estimate of IRD. Pearson product-moment correlation coefficients between the number of mosquitoes captured per locality and the number of rooms sprayed showed that the two variables were either weakly correlated negatively or not correlated (r = −0.23, df = 98, *p* = 0.017 for *An. gambiae* and r = −0.09, df = 98, *p* = 0.360 for *An. funestus*). Moreover, we fitted a binary logistic regression model with a logit link and binomial error structure between the presence of a species in one locality and the number of rooms sprayed. In agreement with correlation tests, the explained deviance was relatively low (17.23%, df = 98, *p* = 0.223 for *An. gambiae* and 4.94%, df = 98, *p* = 0.010 for *An. funestus*), implying that most of the variation in the presence of species in one locality was not related to the sampling effort.

Variance-to-mean ratio was significantly different from unity when indoor resting counts were aggregated at the locality level (*An. gambiae*: *t* = 69.66, df = 99, *p*<0.05; *An. funestus*: *t* = 75.37, df = 99, *p*<0.05). Similarly, Morisita's *Id* exceeded the expectation for a random distribution at this spatial level (*An. gambiae*: *X^2^* = 1079.16, df = 99, *p*<0.05; *An. funestus*: *X^2^* = 1159.60, df = 99, *p*<0.05) (Table 2). More importantly, the two aggregation indices were also significantly different from unity when mosquito counts were aggregated at the house level, indicating that the distribution of the two species remained patchy regardless of the scale of aggregation (Table 2). These indices consistently suggested the presence of some spatial aggregation in the distributions of *An. gambiae* and *An. funestus* at a spatial extent below the 2500 km^2^ study area.

**Table 2:** Non-spatial tests of aggregation.

		Locality level		House level	
Aggregation index	Parameters	*An. gambiae*	*An. funestus*	*An. gambiae*	*An. funestus*
**Variance-to-mean ratio**	Number of sites (n)	100	100	310	310
	Sample mean (*m*)	1.94	0.41	1.93	0.42
	Sample variance (*s^2^*)	6.15	1.36	12.66	1.31
	Variance/mean ratio (*I*)	3.17*	3.31*	6.56*	3.15*
	Distribution	clumped	clumped	clumped	clumped
**Morisita's Index**	Number of sites (n)	100	100	310	310
	Sum of mean IRD (N)	194	41	598	129
	Morisita's Index (*Id*)	2.12*	6.71*	3.88*	6.2*
	Distribution	clumped	clumped	clumped	clumped

* *p*<0.05.

### Spatially explicit tests

Four spatially explicit analyses were carried out and interpreted in combination with visual assessments of abundance maps (Figure 2) to identify spatial patterns of *An. gambiae* and *An. funestus* at two levels of aggregation (locality and house).

#### Global methods

**Figure 3:**
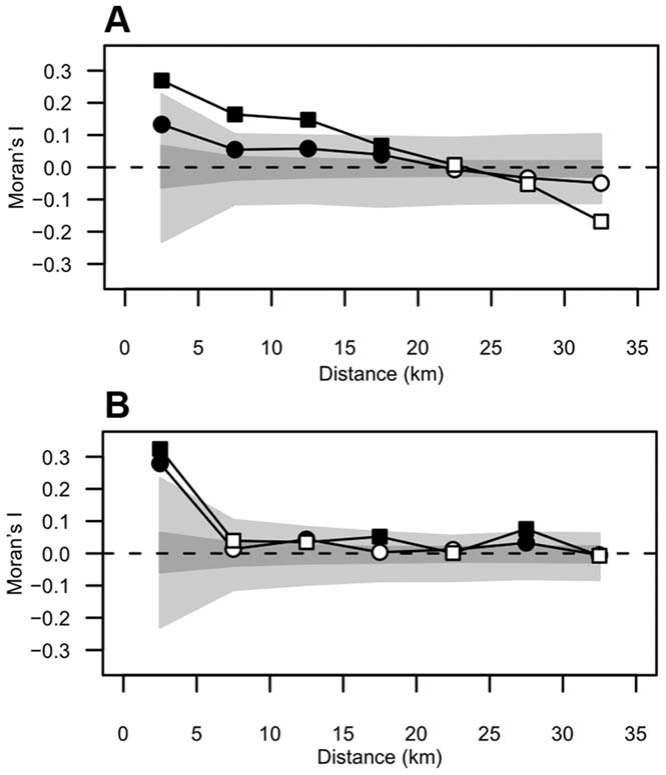
Moran's *I* correlograms of *An. gambiae* (A) and *An. funestus* (B). Circles and squares represent Moran's *I* values at the house level and at the locality level, respectively. Filled symbols indicate statistically significant individual lags (*p*<0.05). Envelopes of 95% confidence intervals are shown in light grey (locality level) and dark grey (house level).

Moran's *I* correlograms of both species had a globally flat form (Figure 3A and 3B). There were positive and statistically significant values of *I* at small distance lags, indicating positive autocorrelation in indoor resting densities between neighboring locations. At the locality level, the highest value of Moran's *I* was recorded at lag 0 (0–5 km) (*I* = 0.27, *p*<0.05 for *An. gambiae* and *I* = 0.32, *p*<0.05 for *An. funestus*). Then, in *An. gambiae*, *I* declined steadily and reached zero at a threshold distance of 20–25 km. Beyond this threshold, autocorrelations were negative or near zero. These results suggest that large clusters of similar values of IRD with a diameter up to 25 km may be found in *An. gambiae* in this region. In the case of *An. funestus*, however, there were only marginal or negative spatial autocorrelations beyond 5 km of distance between localities (Figure 3B), implying that the critical size for clusters of similar values of abundance are considerably smaller in *An. funestus*. This difference is not surprising given the strong divergence of habitat types between the two species: *An. funestus* depends on the presence of much localized permanent breeding sites, whereas *An. gambiae* is a ubiquitous species exploiting collections of water that are widespread spatially. The correlograms of both species displayed the same trends at the house level and at the locality level with statistically significant positive autocorrelations at short spatial lags until the same threshold distances (Figure 3).The curves were similar at the two spatial levels in the case of *An. funestus*, but we noted a certain asymmetry in *An. gambiae* where positives values of *I* were higher while negative values were lower at the locality level compared to the house level.

**Figure 4:**
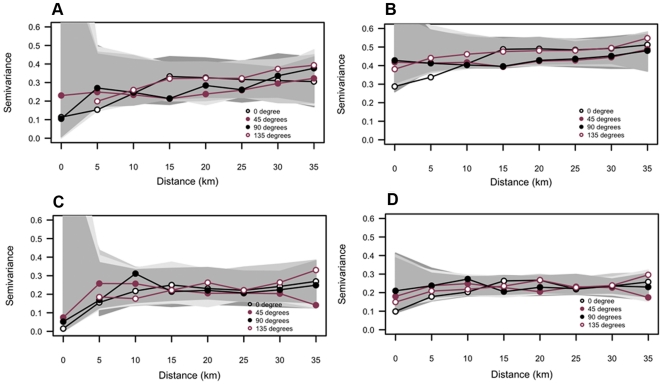
Directional variograms. *An. gambiae*: (A) locality level and (B) house level. *An. funestus*: (C) locality level and (D) house level. Envelopes of minimum and maximum values over 1000 randomizations are shown in grey scale from light (0°) to dark (135°).

Directional variograms confirmed the occurrence of spatial autocorrelation at short spatial lags (Figure 4). On the variograms of *An. gambiae* (Figure 4A and 4B), the nugget effect was high, especially at the house level, implying that an important fraction of information (variability) was not captured with the 5×5 km sampling grid we used, and that there were presumably some other cryptic systematic errors in our data collection and our analytical process. Values of the semivariance varied between 0.1 and 0.5 at the locality level, but no leveling was observed at any distance lag, indicating that the average distance below which the samples are spatially correlated (the range of spatial dependence) could not be identified. There was no anisotropy in the distribution of *An. gambiae* at this spatial level. Each of the four variograms displayed only a very weak spatial trend. Moreover, no trend could be clearly identified in the raw data (Figure 2), and randomization envelopes of variograms overlapped almost entirely in all the four spatial directions (Figure 4A). Plots of the semivariance with values of indoor resting densities aggregated at the house level (Figure 4B) differed from variograms of the locality level. The nugget effect increased considerably, and the slope was reduced at the house level compared to the locality level. At the house level as well, no leveling was observed. Though variograms seemed to display two mild spatial trends at this level of aggregation (one trend following the directions 45° and 90°, and another following the directions 0° and 135°), the randomization envelope also overlapped perfectly, and there was no apparent anisotropy in the distribution of *An. gambiae* at the house level.

On the variograms of *An. funestus* (Figure 4C and 4D), the nugget effect was also high at the house level, and the same conclusion could be drawn from the fact that, most of the fine-scale spatial structure was not captured with the 5×5 km sampling grid we used, especially when mosquito counts were aggregated at the house level. However, contrary to the house level, the semivariance seemed to level off at 5–10 km at the locality level, confirming that the critical size of clusters of similar abundance of *An. funestus* is about 5 km as previously observed with correlograms. At both spatial levels, randomization envelopes overlapped almost perfectly, and there were no apparent trends and no anisotropy in the spatial distribution of *An. funestus* (Figure 2B).

Overall, the two global methods (correlograms and variograms) showed that there was a spatial structure in the distribution of *An. gambiae* and *An. funestus*, and indicated different boundaries for critical distances of aggregation for the two species. As a result, the use of local spatial statistics should further identify important local patterns in the distribution of the two species.

#### Local methods

**Figure 5:**
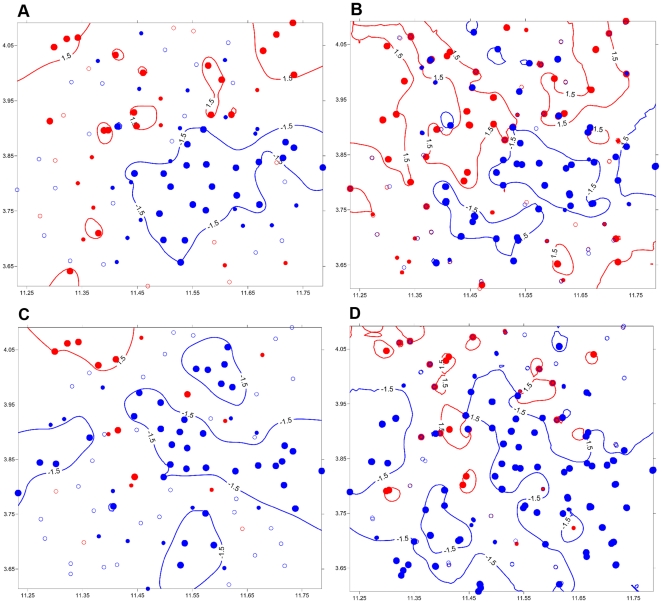
Maps of SADIE clustering indices. *An. gambiae*: (A) locality level and (B) house level. *An. funestus*: (C) locality level and (D) house level. Each sample unit is characterized by a positive (red circles) or a negative (blue circles) clustering index. Small open circles: absolute value of clustering below expectation (1); small filled circles: sample units with clustering that exceeds expectation (<−1 or >1); large filled circles: sample unit with high clustering indices (<−1.5 or >1.5).

Table 3 summarizes the results of SADIE analyses, and more detailed information on characteristics of spatial clusters is mentioned in Table 4. A strong aggregation of *An. gambiae* counts at the locality level was confirmed by a large and significant value of *Ia* (*Ia* = 1.644, *Pa*<0.0007). The clustering indices of this species at this spatial level was characterized by a non significant clustering into a single large gap (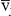 = −1.448, *P*_j_>0.05) comprising twenty-two sample units and extending about 25 km from the center towards the eastern side of the study area, as well as a significant clustering into three big and seven small patches (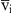 = 1.704, *P*_i_ = 0.022) (Figure 5A). All these patch clusters encompassed 20 localities with IRD varying from 1.67 to 10.6 (mean ± standard error: 3.56±2.37). At the house level, the pattern of aggregation of *An. gambiae* was stronger, with a higher and statistically significant value of *Ia* (*Ia* = 1.966, *Pa*<0.0002). At this level, the spatial pattern was characterized by three large and four small gaps (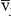 = −1.904, *P*_j_ = 0.0002) adjacent to three large and four small patches (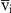 = 1.783, *P*_i_ = 0.0008) (Figure 5B). The mean ± standard error of IRD was 3.07±3.91 and 0.08±0.26 in all patches and all gap clusters, respectively.

**Table 3:** Summary of SADIE analyses.

Species	Spatial level	*I*a	*P*a		*P_j_*		*P_i_*
*An. gambiae*	locality	1.644	<0.0007	−1.448	0.0900	1.704	0.0220
	house	1.966	<0.0002	−1.904	0.0002	1.783	0.0008
*An. funestus*	locality	1.464	<0.0800	−1.495	0.0069	1.506	0.0060
	house	1.980	<0.0002	−1.972	0.0002	1.981	0.0002

**Table 4:** Characteristics of SADIE and LISA clusters.

Spatial level	Species	Characteristics of clusters	SADIE clusters		LISA clusters			
			Patch	Gap	High-High	High-Low	Low-High	Low-Low
**Locality**	***An. gambiae***	Range IRD	1.67–10.6	0–1.36	8.25–10.75	5.2	0.4	0–0.25
		Average±SE IRD	3.56±2.37	0.34±0.37	9.83±1.42	5.2	0.4	0.03±0.09
		Sampling units	20	23	4	1	1	8
	***An. funest*** *us*	Range IRD	0.71–5	0–0.45	0.14–1.8	2.7–6.5	/	/
		Average±SE IRD	2.34±1.73	0.07±0.13	2.42±1.97	4±1.69	/	/
		Sampling units	8	37	4	5	/	/
**House**	***An. gambiae***	Range IRD	0.5–27	0–2	1–33	1–27	0–0.5	0.33
		Average±SE IRD	3.07±3.91	0.08±0.26	13.42±13.65	12.83±8.4	0.17±0.29	0.33
		Sampling units	83	67	5	7	3	1
	***An. funestus***	Range IRD	0.5–7	0–0.4	0–7	1.67–6.5	0	/
		Average±SE IRD	2.16±1.88	0.02±0.08	3.32±1.85	4.29±2.03	0±0	/
		Sampling units	32	136	20	4	4	/

/ No cluster; SE standard error.

Concerning *An. funestus* whose abundance in the study area was notably less than that of *An. gambiae*, the index *Ia* showed no evidence of aggregation at the locality level (*Ia* = 1.464, *Pa*>0.05). However, there was significant clustering in patches and gaps (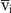 = 1.506, *P*_i_ = 0.0060; 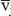 = −1.495, *P*_j_ = 0.0069). The clustering pattern was characterized mainly by four big gaps among which a dominant gap cluster comprising about 22 sampling sites and only one patch cluster located in the North-West of the area (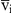 = 1.981, *P*_i_<0.05; 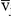 = −1.972, *P*_j_<0.05) (Figure 5C). Consistently, average IRD within patches and gap clusters were also significantly less than those observed in *An. gambiae* clusters (Table 4). The global spatial pattern of *An. funestus* was different at the house level: there was strong evidence of spatial non-randomness as shown by the large and significant value of *Ia* (*Ia* = 1. 980, *Pa*<0.0002). Clustering indices further supported the presence of four big gaps; particularly, a dominant gap cluster spanning almost 40 km from the center towards the south-east of the study area (Figure 5D). This gap comprised about 40 sampling sites, and was by far the largest recorded in the study area. By contrast, the significant average patch clustering index was associated with the presence of about twelve very small patches scattered in the northern part of the area. IRD varied from 0.5 to 7 within patch clusters and from 0 to 0.4 in gap clusters (Table 4). In general, as previously revealed by the correlograms and variograms, large clusters of locations with greater than average IRD with a diameter around 25 km could be found in *An. gambiae*, but clustering of *An. funestus* occurred in the form of small patches with a diameter lower than 5 km. The indices v_i_ and v_j_ encapsulate spatial and not numeric information; hence, a lack of relationship between the magnitude of counts and the degree of clustering can be observed. Yet, maps of clustering indices and abundances were very consistent. The region of low abundance of *An. gambiae* in the center of the abundance map (Figure 2) overlapped with the big gap clusters on contour maps (Figure 5). Similarly, large counts of *An. gambiae* were recorded mostly in sample sites situated in the upper part of the study area, overlapping with the three big patch clusters of the grid. Likewise, the few small patches identified in *An. funestus* distribution coincided approximately with sample sites with considerably high counts of this species.

**Figure 6:**
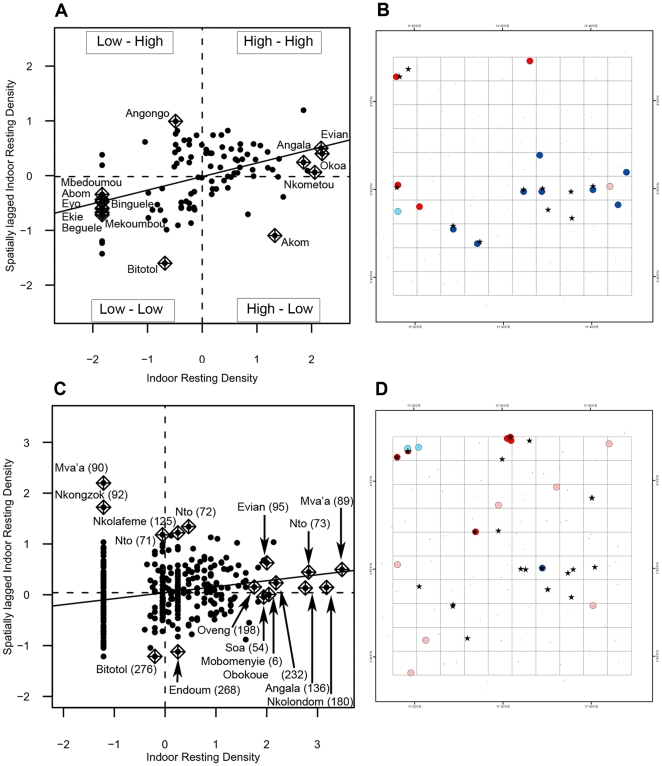
LISA results for *An. gambiae*. (A) and (C) are Moran scatter plots at the locality and at the house level, respectively. The name of the locality and the house number (in brackets) with large contributions to autocorrelation are displayed. (B) and (D) depict the locations of significant local Moran's *I* statistics and the type of spatial association between neighboring locations in sampling units with large contributions to the global autocorrelation. (A): locality level and (B): house level. ∧ significant (*p*<0.01); bright red: *High-High*; light red: *High-Low*; deep blue: *Low-Low*; light blue: *Low-High*.

**Figure 7:**
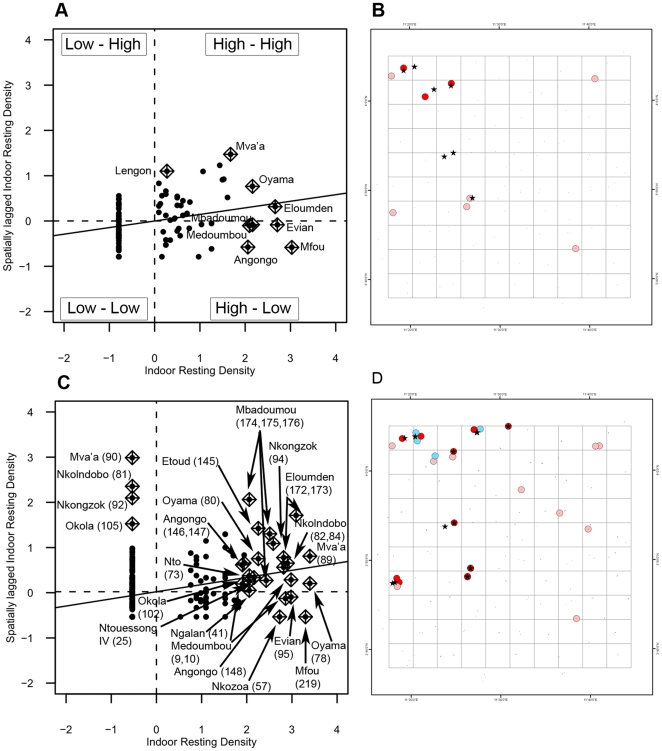
LISA results for *An. funestus*. (A) and (C) are Moran scatter plots at the locality and at the house level, respectively. The name of the locality and the house number (in brackets) with large contributions to autocorrelation are displayed. (B) and (D) depict the locations of significant local Moran's *I* statistics and the type of spatial association between neighboring locations in sampling units with large contributions to the global autocorrelation. (A): locality level and (B): house level. ∧ significant (*p*<0.01); bright red: *High-High*; light red: *High-Low*; deep blue: *Low-Low*; light blue: *Low-High*.

Figures 6 and 7 show the Moran scatter plots and maps of spatial locations with statistically significant clustering (*p*<0.01), as well as sampling units that have a great influence on the global autocorrelation in LISA analyses. Characteristics of local clusters are summarized in Table 4. Significant local clustering of indoor resting density was detected in 11 localities in *An. gambiae* and in only six localities in *An. funestus*. The number of significant clusters increased, of course, at the house level (20 in *An. gambiae* and 11 in *An. funestus*), but this rise was not strictly proportional to the increase in the number of sampling units between the two scales of aggregation. On the Moran scatter plot of *An. gambiae*, at the locality level, sampling units were well distributed in the four quadrants of the scatter plot, and the four regimes of spatial association between spatial units (*High-High*, *High-Low*, *Low-High*, *Low-Low*) were represented (Figure 6A). Among sampling units that have high influence on the global autocorrelation at the locality level, *Low-Low* (cold spots) and *High-High* (hot spots) represented the two dominant types of association, with 8 and 4 sampling units respectively (Table 4). At the house level, in contrast, the dominant types of interaction among spatial units were *High-High* (5 units) and *High-Low* (7 units), highlighting the fact that there are important variations among houses and a fine-scale spatial structure that occur below the locality level. This idea is strengthened by the fact that certain houses belonging to the same locality appear in different quadrants of the Moran scatter plot (Figure 6B). In hot spots of *An. gambiae*, IRD varied from 8.25–10.75 at the locality level and from 1 to 33 at the house level (Table 4).

In *An. funestus*, at both aggregation levels, there were mainly *High-High* and *High-Low* associations between neighboring spatial locations (Figure 7A and 7C), confirming that the distribution of adults of this species is characterized by very small local clusters of individuals that occur around characteristic breeding sites. Though the two aggregation levels presented the same types of association between spatial locations, sub-locality structure could be observed as a few houses of the same locality appeared in different quadrants of the scatter plot at the house level. Hot spots of this species comprised 4 spatial units at the locality level and 20 at the house level, and the mean ± standard error of IRD within these hot spots were 2.42±1.97 at the locality level and 3.32±1.85 at the house level, respectively (Table 4).

When comparing LISA and SADIE clusters, by a visual examination and quantitative assessment, there were substantial differences in the number, the size and the spatial distribution of clusters detected by each of the two methods (Table 4). For instance, the number of SADIE patches (spatial points with 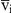
>1.5) was two to sixteen times greater than the number of sites classified as hot spot by LISA analyses. Nevertheless, despite this significant inter-method variation, it is interesting to notice that almost all the sampling units that were considered hot spot or cold spot clusters by the Local Moran's *I* were geographically embedded respectively in patch and gap clusters identified by SADIE analyses (Figure 5, 6 and 7). The parameterization of the two methods is not strictly analogous due to unique characteristics of both methodologies. Moreover, we have applied LISA in a manner that could detect clusters only at point locations while SADIE enables to map spatial extents of clusters in two-dimensional space. However, the combination of these two local tests provides a useful comparison and potentially greater evidence for clustering patterns.

## Discussion

Recent methodological advances in spatial statistics combined with the ready availability of inexpensive and powerful desktop geographic information systems have strongly promoted the use of spatially explicit methods in exploring spatial patterns of point-referenced counts in epidemiology and ecology [24], [25]. In the present study, we have conducted a methodological and comparative evaluation to assess the benefits of using a variety of techniques for geographic pattern detection in *Anopheles* mosquito counts, with the variable “indoor resting density” as an example. In general, regarding the pattern of spatial distribution of a species, the main question is to know whether individuals or populations are arranged in a random, regular or aggregated manner in space. When the distribution exhibits a spatial structure (non-random distribution) in a study area, it is essential to be able to map and characterize the spatial clusters of individuals. We have first applied two non-spatial methods, the Morisita's index and the variance-to-mean ratio, to test the spatial aggregation of the two numerically dominant malaria vectors in our study area. The two methods indicated that the distribution of *An. gambiae* an *An. funestus* was patchy in this area, regardless of the scale at which mosquito counts were aggregated. We have also used several spatial analysis tools to assess the spatial structure and analyze the spatial clusters of individuals of the two *Anopheles* species. Our results showed that these combined analyses provided a more comprehensive diagnostic, with more consistent interpretations than could have otherwise been obtained with any one statistical approach alone. Correlograms and variograms suggested the existence of spatial structure in the distribution of *An. gambiae* and *An. funestus* in the study area, which resulted in the occurrence of spatial autocorrelation between neighboring spatial locations at certain distance lags. These two global methods also estimated the threshold distance for the spatial extent of major clusters of similar values of abundance. Variograms revealed that the resolution of the sampling grid we used was not fine enough to capture an important part of the variability of indoor resting densities. SADIE and LISA indicators identified hot spots and cold spots of abundance that were mapped and characterized. Our analyses also showed that a spatial structure may occur at the sub-locality level, and underscored the benefits of multi-scalar approaches in assessing geographic patterns of *Anopheles* distribution [54]. Spatially explicit tests of spatial aggregation corroborated the results provided by the two non-spatial methods we used, with the exception of *An. funestus* data aggregated at the locality level. In this dataset, Morisita's index and variance-to-mean ratio showed a clumped distribution, whereas SADIE analyses indicated that there was no evidence of spatial aggregation. This disagreement between the two sets of methods is often observed, leading to a certain controversy in the interpretation of spatial aggregation [55]. However, although non-spatial methods are simpler to implement, in our study, using them did not provide any additional useful information than using spatially explicit methods alone.

A variety of spatial statistical techniques are designed for uncovering spatial clusters of disease prevalence in epidemiology. Most of these methods are now readily included in common Geographic Information System software packages, as well as in various standalone programs. These programs include, for instance, GeoDa, SaTScan, Crimestat, Clusterseer and many packages of the open source statistical program R [23], [25], [51], [56]. There is an important literature that introduces, classifies and compares some of the most common methods implemented in these computational tools and provides insights into the tradeoffs among different approaches [25], [56], [57]. The majority of these spatial statistical techniques can also be applied to any count data, including spatially referenced censuses of species. Nevertheless, Perry *et al.* [14] have tested and compared an important array of techniques encompassing the correlogram, the variogram, spatial interpolation, SADIE and Ripley's K, that can be used specifically for spatial analyses of species distribution. The main recommendation that emerges from all these comparative investigations on spatial analysis tools is that the use of several global and local methods at the same time should provide the greatest reliability. It is also advised to use simple visualization techniques for initial analysis, before selecting the methods that are appropriate for the data type, and that answer the specific questions of interest.

On the other hand, the interpretation of spatial statistical analyses requires a lot of caution because there are several caveats that apply to many, if not all such analyses. The ignorance of these caveats can sometimes lead to weakened or erroneous conclusions [25]. First, each type of approach requires the use and the specification of spatial relationships among the spatial units. This relationship is usually specified in the form of spatial weights that are designed in very diverse manners, and the results of analyses can thus differ strongly depending on how the spatial weights have been specified [47], [51], [57]. Moreover, all techniques for spatial pattern analysis are founded on assumptions that are sensitive to the data types, the scales of observation and sampling designs [34], [52]. These caveats make it difficult to standardize a relevant methodological framework, from different empirical observations, in a given operational context. There are also limitations pertaining to each individual method that should be taken into account when interpreting the results [14], [33]. While employing a variety of techniques doesn't remove individual flaw of each method, it does illuminate different aspects of spatial patterns, thereby providing a more accurate description of spatial heterogeneity [26], [33], [57]. Spatial statistical analyses have been used in several studies to infer the spatial patterns of mosquitoes from point count collections of a set of sampling locations. Ribeiro *et al.* [58] used the kriging to estimate the spatial and temporal variation of *Anopheles* species densities at the level of one village in Ethiopia. Moran's *I* was applied by Jacob *et al.* [30] to analyze the spatial structure of the field-sampled count data of *Aedes albopictus* and *Culex quinquefasciatus* in ten locations within three adjacent neighborhoods of an 8 km^2^ grid. Li *et al.* [29] also employed this global test to identify the extent of spatial autocorrelation between nearby samples in a 4×4 km study area in Kenya. Ryan *et al.* [27] used both Moran's *I* and kriging to examine the spatial patterns of four different mosquito species in Australia. In Kenya, Kelly-Hope et al. [31] investigated on the spatial distribution of the relative contribution of three malaria vectors (*An. gambiae sensu stricto*, *An. arabiensis* and *An. funestus*) to annual malaria transmission with the global Moran *I* test and the Getis-Ord Gi* statistic. de Souza *et al.* [32] also used the two statistics for spatial analysis of *An. gambiae* distribution across the country in Ghana. Finally, in Zhou *et al.* [28], correlography was used to determine the spatial autocorrelation in adult mosquito abundance and Getis-Ord Gi* index employed to define focal abundance clusters. Though these studies provided relevant descriptions of spatial attributes, using only one or two spatial analysis tools may be insufficient to effectively describe the spatial structure, given the flaws pertaining to each method, the error linked with the specification of spatial weights and the sensitivity to sampling design as we mentioned earlier. For example, significant local clustering may occur where global statistics do not provide evidence of spatial autocorrelation. By contrast, there may be a strong and significant indication of global autocorrelation where local patterns are totally random, especially in large datasets [23].

There are some limitations to our study that are worth noting. First, our analyses did not address the temporal aspect of spatial heterogeneity. The data collection took place only in one year, and we were unable to gauge the persistence of the spatial structure and clusters in time. As a result, our approach is appropriate to provide mostly a first detailed snapshot rather than to uncover consistent patterns with long-term stability. We underscore the need for spatial analyses such as those presented here to be repeated during subsequent years so as to address both spatial and temporal dynamics. Secondly, variograms, in particular, indicated that the 5 km sampling resolution we used was probably coarse. Ideally, a spatial sampling design should be based on clear criteria tailored for the particular application. We chose our sampling resolution based on the maximum dispersal distance of adult *Anopheles* mosquitoes and the necessity to have at least one locality to survey within each cell of our sampling grid. However, the maximum flight distance of African *Anopheles* mosquitoes is not very clear, and many distances ranging from 1 to 5 km have been estimated [9], [38]. Ideally, we should have quantitatively analyzed the impact of changing the cell size of the sampling grid on our results in order to propose a more accurate window size for field mosquito collections. In addition, the sampling resolution required for studying spatial patterns of *Anopheles* mosquitoes in operational activities should probably target spatial resolutions below what we used here. Another potential source of bias may arise from the uneven allocation of sampling effort caused by the variation of the number of houses sampled per locality, but correlations tests showed that this has little confounding effect on the estimates of indoor resting densities. Nevertheless, rather than an attempt to establish rigid guidelines for spatial analyses, we mostly underlined some key issues in sampling and spatial aggregation of *Anopheles* mosquitoes, with the hope that our study can contribute to the design of approaches that are based on a better understanding of methodological and analytical techniques.

The practical application of spatially explicit analyses of anopheline mosquitoes is to support evidence-based decisions in malaria control activities. Indeed, spatial and temporal analyses of indicators like local epidemiological data, vector distribution and behavior, insecticide resistance status and sporozoite rates are pivotal prerequisite to successful malaria control strategies. Overall, malaria control campaigns implemented in many African countries are based on integrated strategies encompassing several intervention methods at the community level. The control operations are usually clustered in space, especially when allocated resources are limited [2], [9]. Targeted malaria control has been successfully implemented to reduce malaria transmission in several countries across the continent [10], [11], [12]. In this intervention procedure, treatments are provided in priority to limited areas that are identified based on thresholds of transmission (high risk areas). Although the relationship between the transmission intensity and vector abundance is not linear, the study of the spatial structure of vector species and their habitats can provide relevant additional key variables in the selection of rate-limiting or priority areas, and the design vector control methods that integrate both transmission levels and threshold of other entomologic parameters among decision tools [10], [12]. Prior knowledge of the spatial and temporal dynamics of the abundance of *Anopheles* adults is particularly useful for malaria control measures such as indoor residual insecticide spraying, the distribution of long-lasting insecticide-treated nets or environmental management [2], [59], [60]. For instance, in indoor residual spraying which is a well-established control method for malaria mosquitoes integrated in malaria control programs in many African countries, the size of the operational area depends on local circumstances and is influenced by the distribution of malaria vectors, the distance from important breeding sites, the flight range of vectors and demographic factors [12], [61], [62]. Regular mass spraying of all human dwellings is technically unfeasible, especially when the treatment area is large, and the effectiveness of this method is closely linked to the capacity to optimally delimit target houses or areas within an operational region. Spatial patterns of relevant local attributes that are necessary to take such decisions can be effectively addressed with applied spatial statistics used in conjunction with geographic information systems. Significant efforts are already being undertaken to integrate geographic information systems in the planning and implementation of indoor residual spraying in national malaria control programs [63]. We have shown in this study that spatial analysis tools are effective to infer point and surface patterns of entomologic parameters from point collections at selected sampling locations. They can also help identify how many villages or how many houses lie within significant clusters of abundance of a particular vector species. Therefore, spatial analyses of indicator variables like species diversity and distribution, vector abundance, insecticide resistance rate and entomological inoculation rate can ultimately help guide strategic decisions to more efficiently target vector control activities.

The characterization of spatial patterns can also assist in identifying species-habitat relationships and associations between vectors occurrence or abundance and key environmental variables. Recent efforts have been made to model habitats of the most important African malaria vectors [3], [39], [64], [65]. The spatial statistical tests can be integrated into these efforts to explore in more detail the species-environment relationships of *Anopheles* species. Biological factors underlying the spatial distribution of species are numerous, but the variation between the spatial structure of *An. gambiae* and *An. funestus* demonstrated by our analyses is at least partially driven by the strong difference between larval habitats of the two species. *An. gambiae* has more opportunistic breeding habits while *An. funestus* adults show a strong tendency to be aggregated in houses located on the edges of their typical breeding sites [54]. In our study, it was difficult to relate patches and gap clusters of *An. gambiae* to precise environmental features, but we found for instance that the urban neighborhoods of Yaoundé were embedded in the most important gap cluster of *An. funestus* which is less adapted to urban areas than *An. gambiae* [3]. Another benefit of spatially explicit analyses may be to assist in sampling designs of mosquito collections. The observed patterns of species abundance affect the sampling design [5], [66]. Diversities and densities of malaria vectors vary widely in space and time, making it particularly difficult to predict the sampling effort necessary for accurate estimates of mosquito abundance. Most often, statistical methods used to calculate the sampling effort required to attain pre-established levels of precision rely on prior knowledge of the degree of spatial aggregation in one population. In practical conditions, this aggregation is usually examined by non-spatial tests such as the negative-binomial distribution or the Taylor's power law whose limitations have been discussed [6], [7], [8], [14]. Moreover, studies on estimates of entomologic parameters most frequently assumed mosquito densities for a limited number of houses within a village to be representative of the whole village. This may introduce some errors into the estimates, given the level of variation among houses within one village highlighted by our study and previous investigations [54]. However, how precisely the sampling schemes can be accommodated to account for spatial heterogeneity of populations is a great problem for classical statistical tests which cannot be solved even by employing spatial statistical tools. Nevertheless, spatially explicit analyses provide a more detailed description of spatial patterns and a more credible identification of dispersion profiles. As a result, models integrating sampling precision and sampling effort could be adapted to spatially explicit tests of spatial aggregation to improve the accuracy in estimates of population parameters of *Anopheles* mosquitoes.
